# MR Biomarkers of Degenerative Brain Disorders Derived From Diffusion Imaging

**DOI:** 10.1002/jmri.27019

**Published:** 2019-12-13

**Authors:** Christina Andica, Koji Kamagata, Taku Hatano, Yuya Saito, Kotaro Ogaki, Nobutaka Hattori, Shigeki Aoki

**Affiliations:** ^1^ Department of Radiology Juntendo University Graduate School of Medicine Tokyo Japan; ^2^ Department of Neurology Juntendo University School of Medicine Tokyo Japan; ^3^ Department of Radiological Sciences Tokyo Metropolitan University, Graduate School of Human Health Sciences Tokyo Japan

**Keywords:** bi‐tensor diffusion tensor imaging, single‐tensor diffusion tensor imaging, diffusion kurtosis imaging, neurite orientation dispersion and density imaging, neurodegenerative diseases

## Abstract

The incidence of neurodegenerative diseases has shown an increasing trend. These conditions typically cause progressive functional disability. Identification of robust biomarkers of neurodegenerative diseases is a key imperative to facilitate early identification of the pathological features and to foster a better understanding of the pathogenetic mechanisms of individual diseases. Diffusion tensor imaging (DTI) is the most widely used diffusion MRI technique for assessment of neurodegenerative diseases. The DTI parameters are promising biomarkers for evaluation of microstructural changes; however, some limitations of DTI restrict its wider clinical use. New diffusion MRI techniques, such as diffusion kurtosis imaging (DKI), bi‐tensor DTI, and neurite orientation density and dispersion imaging (NODDI) have been demonstrated to provide value addition to DTI for evaluation of neurodegenerative diseases. In this review article, we summarize the key technical aspects and provide an overview of the current state of knowledge regarding the role of DKI, bi‐tensor DTI, and NODDI as biomarkers of microstructural changes in representative neurodegenerative diseases including Alzheimer's disease, Parkinson's disease, amyotrophic lateral sclerosis, and Huntington's disease.

**Level of Evidence:**

5

**Technical Efficacy Stage:**

2 J. MAGN. RESON. IMAGING 2020;52:1620–1636.

THE PREVALENCE OF NEURODEGENERATIVE DISORDERS is increasing, in part due to increased lifespan. According to the United Nations report on world population aging (2017),^1^ the estimated global population aged ≥60 years in 2017 was 962 million and the number is projected to double by 2050. Aging is the main risk factor for the development of neurodegenerative diseases and the associated burden of disease has high cost implications.^2^ Therefore, development of effective disease‐modifying or neuroprotective therapies for neurodegenerative diseases is a key research imperative^3^; however, this requires an in‐depth understanding of the etiopathogenesis of the individual diseases. In addition, there is a need to develop sensitive biomarkers to identify the pathological features of neurodegenerative diseases at an early stage so that the treatment can be started prior to significant cell loss.

Diffusion magnetic resonance imaging (MRI), which measures the random motion of water molecules in tissues along a specified direction, is a promising approach for noninvasive characterization of tissue microstructure. Currently, diffusion tensor imaging (DTI)^4^ is the most widely used diffusion MRI technique for the study of neurodegenerative diseases. The metrics obtained from DTI, such as fractional anisotropy (FA) and mean diffusivity (MD), are potential biomarkers of brain abnormalities in patients with neurodegenerative diseases.^5^ Nonetheless, the limitations of DTI have been well described and are elaborated below.

Recent developments in diffusion MRI have addressed some of the limitations of DTI. Diffusion kurtosis imaging (DKI),^6^, ^7^ bi‐tensor DTI,^8^ and neurite orientation dispersion and density imaging (NODDI)^9^ are increasingly used for the evaluation of neurodegenerative diseases that can be performed within a clinically feasible time frame. These approaches can be performed using the standard MRI scanners within a clinically feasible time frame. In short, DKI represents an extension of DTI that allows the quantification of non‐Gaussian water diffusion properties in the brain^6^, ^7^; bi‐tensor DTI was developed to estimate and remove the signal contributions from cerebrospinal fluid (CSF) and apparent free‐water components from the estimated diffusion tensor of tissue^8^; the biophysical tissue models of NODDI may provide specific biomarkers of brain microstructural changes, such as the density and orientation dispersion of neurites.^9^ Table 1 summarizes the properties of each of these approaches. Although these approaches also have their own limitations, these have been shown to provide added value to DTI in the evaluation of neurodegenerative diseases.

**TABLE 1:** Summary of the Characteristics of Various Diffusion MRI Modalities

Method	Minimum/typical data requirements	Measures	Abbreviations	Parameters related to
DTI	1 b = 0; 6 b = 1000 s/mm^2^ / 1 b = 0; 30 b = 1000 s/mm^2^	FA	Fractional anisotropy	Overall directionality of water diffusion within the brain tissue
		MD	Mean diffusivity	Magnitude of isotropic diffusion within the brain tissue
		AD	Axial diffusivity	Magnitude of isotropic diffusion within the brain tissue along the direction of maximal diffusion
		RD	Radial diffusivity	Magnitude of isotropic diffusion within the brain tissue perpendicular to the direction of maximal diffusion
DKI	1 b = 0; 6 b = 1000; 15 b = 2000 s/mm^2^ / 1 b = 0; 30 b = 1000; 30 b = 2000 s/mm^2^	MK	Mean kurtosis	Microstructural complexity or heterogeneity within the brain tissue
		AK	Axial kurtosis	Microstructural complexity or heterogeneity within the brain tissue along the direction of maximal diffusion
		RK	Radial kurtosis	Microstructural complexity or heterogeneity within the brain tissue perpendicular the direction of maximal diffusion
Bi‐tensor DTI	1 b = 0; 6 b = 1000 s/mm^2^ / 1 b = 0; 64 b = 1000 s/mm^2^	FW	Fractional volume of free‐water	Volume fraction of extracellular free‐water within the brain tissue
		FA_T_	Free‐water corrected FA	
		MD_T_	Free‐water corrected MD	
		AD_T_	Free‐water corrected AD	
		RD_T_	Free‐water corrected RD	
NODDI	1 b = 0; 30 b = 700; 60 b = 2000 s/mm^2^ / 14 b = 0; 8 b = 300; 32 b = 700; 64 = 2000 s/mm^2^	NDI	Neurite density index	Density of neurites (axons and dendrites) based on intracellular diffusion
		ODI	Orientation dispersion index	Dispersion of neurites (axons and dendrites) in the intracellular compartment
		ISO	Isotropic volume fraction	Volume fraction of isotropic diffusion

In this review we discuss the key technical aspects and provide an overview of the current state of knowledge regarding the role of these more advanced diffusion MRI approaches. We discuss their potential to provide biomarkers of microstructural changes in neurodegenerative diseases including Alzheimer's disease (AlzD), Parkinson's disease (PD), amyotrophic lateral sclerosis (ALS), and Huntington's disease (HD). Since the use of DTI for evaluation of neurodegenerative diseases has been extensively reviewed, we will only briefly touch on the topic of DTI.

## Diffusion MRI Techniques

### *Diffusion Tensor Imaging*

The principles of DTI and its basic concepts have been extensively reviewed in the literature (see references 10, 11 for a more detailed description). DTI indices such as FA, MD, radial diffusivity (RD), and axial diffusivity (AD) characterize the orientation distribution of the random movement of water molecules, diffusion magnitude, diffusional directionality along the axon, and diffusional directionality perpendicular to the axon, respectively (Table 1).^12^

As described above, the DTI parameters have limited clinical utility due to some limitations. First, DTI ignores the non‐Gaussian properties of biological tissues,^4^ while tissue heterogeneity and biological restrictions in the tissue microstructure (such as cell membranes and myelin sheets) are known to cause non‐Gaussian distribution of water diffusion.^6^, ^7^ Second, the assumption of a single‐tissue compartment per voxel such that partial volume effect averaging in a voxel due to extracellular free water^12^ can introduce bias in the interpretation of DTI indices, such as reduced FA and increased MD.^13^ Third, the lack of biological specificity of DTI measures.^10^ For example, a decrease in FA accompanied by increased MD may be attributed to alleviation of neuronal injury or demyelination.^14^, ^15^ AD and RD are shown to be sensitive to axonal injury/degeneration and the degree of myelinization, respectively.^16^ However, these interpretations have also been contested in the literature.^17^ Also, these parameters may provide an acceptable approximation if the voxel includes a healthy fiber bundle, which determines the diffusion characteristic of the voxel. Such an approach, however, can lead to misinterpretation of the results in the case of low signal‐to‐noise ratio, the presence of crossing fibers, or a decrease in anisotropy induced by the underlying pathology.^18^

### *Diffusion Kurtosis Imaging*

DKI was proposed as a mathematical extension of DTI. Kurtosis is a dimensionless measure that quantifies the non‐Gaussian distribution of water diffusion in a voxel.^6^, ^7^ To describe this non‐Gaussian diffusion behavior, kurtosis was introduced as the fourth tensor of distribution,^19^ with the following equation^20^:

(1) $$1n\left[\frac{S\left(b\right)}{S_{0}}\right]=-bD_{\mathrm{app}}+\frac{1}{6}b^{2}D_{\mathrm{app}}^{2}K_{\mathrm{app}}$$

where *D*_*app*_ and *K*_*app*_ are the apparent diffusion coefficients and kurtosis along a certain diffusion direction, *S(b)* is the diffusion‐weighted signal along that direction with a certain b value, and *S*_0_ is the nondiffusion‐weighted signal.

The kurtosis parameters such as mean (MK), the average of the diffusion kurtosis along all the diffusion directions; axial kurtosis (AK), the kurtosis along the axial direction; and radial kurtosis (RK), the kurtosis along the radial direction are suitable for evaluating neuronal integrity in white matter (WM) regions with complex arrangements, including in areas with crossing fibers (Table 1).^21^ Moreover, DKI extends the conventional DTI measures by detecting microstructural changes not only in WM (anisotropic tissues), but also in gray matter (GM; isotropic tissues) because it is independent of the spatial direction of the structures.^7^, ^22^ The higher the diffusion kurtosis, the more the water molecule diffusion deviates from the Gaussian distribution, which is indicative of a more restricted diffusion environment.^23^ Thus, DKI may be superior to conventional DTI with respect to sensitivity for the detection of pathological changes in neuronal tissues. However, changes in the DKI parameters are difficult to interpret because of their poor specificity.^24^ Another limitation of DKI is that the model is more complex than DTI (a minimum of two nonzero b‐values and at least 15 diffusion directions are required [Table 1]); thus, the acquisition time is longer compared with that of DTI. However, with a shorter imaging protocol (ie, 7‐min protocol), the DKI parameters can be variable across brain regions.^25^ Nevertheless, the test–retest reproducibility of DKI metrics was shown to be comparable to that of DTI (coefficient of variation ≤4.5%).^26^ However, if only the MK is of clinical interest instead of the full tensors, then a fast DKI acquisition can be performed within 1–2 minutes.^20^

### *Bi‐tensor DTI*

Free water is defined as the water molecules that do not flow and are not restricted. In the human brain, free water is present as CSF in the ventricles and around the brain parenchyma. Free water may also accumulate in the extracellular space in the brain parenchyma because of brain pathologies such as tumors, trauma, and neuroinflammation.^8^, ^27^

Bi‐tensor DTI enables the differentiation between alterations in the tissues themselves, as measured by the free water‐corrected DTI indices (referred as FA_T_, MD_T_, RD_T_, and AD_T_) and extracellular free water changes, as measured by the fractional volume of free water (referred as FW) (Table 1).^8^ This is performed by adopting a two‐compartment model and fitting two tensors into the diffusion data: one anisotropic (tissue compartment, *C*_*tissue*_) and the other isotropic with diffusion characteristics of free water (*C*_*water*_).^8^ The maps of bi‐tensor DTI were calculated by fitting the following model in each voxel^8^:

(2) $$1=A_{\mathrm{bi}-\text{tensor}}\left(D,f\right)=C_{\text{tissue}}+C_{\text{water}}=fA_{\text{tissue}}\left(D\right)+\left(1-f\right)A_{\text{water}}$$

where *A*_*bi–tensor*_ is the voxelwise modeled attenuation vector that has an entry for each diffusion orientation and *A*_*tissue*_ and *A*_*water*_ are the represented model of attenuation vectors. The scalar *f* is the fractional volume of the tissue compartment (0 < *f* < 1), and similarly (1‐*f*) is the fractional volume of free water. The FW compartment has a fixed diffusivity of 3 × 10^‐3^ mm^2^/s (the diffusion coefficient of the FW at body temperature).^28^

The advantage of this model is that the method requires a single‐shell DTI acquisition and can be easily merged with the existing DTI pipelines.^8^ Compared to the single‐tensor DTI model, the bi‐tensor DTI was shown to exhibit better tissue specificity for the characterization of human WM (both in healthy and in diseased states)^13^ and a greater sensitivity for the detection of microstructural changes.^29^ Bi‐tensor DTI has also been shown to reduce the test–retest reproducibility errors of DTI metrics^29^ and improve DTI‐based tract reconstruction.^8^ Another potential advantage is the extraction of an FW map that might be used as a biomarker of neuroinflammation.^30^, ^31^ Unfortunately, it is not feasible to histologically confirm FW as a marker of neuroinflammation, as it is an active physiological process that is not observed in fixed samples.^32^ However, a recent study has demonstrated the correlation between FW obtained using bi‐tensor DTI and positron emission tomography (PET) imaging with 18‐kDa translocator protein‐binding ligands, a measure of neuroinflammation.^33^

However, the bi‐tensor DTI model does have some limitations. First, the assumption that there is no exchange of water molecules between the compartments. In this context, precaution has to be taken with edema, since it might be correlated with the changes in tissue permeability. An increase in the exchange rate is expected to cause a bias in the estimation of FA, and, therefore, a bias in the FA values of the tissue compartment.^8^ The same limitation applies in GM, wherein the cell bodies are more permeable than the myelin sheets of the fiber bundles.^8^ Another limitation of bi‐tensor DTI is that the model is derived from a bi‐tensor model, which consists of an FW compartment and a single fiber population. However, an estimated 66–90% of brain WM voxels contain at least two fiber bundles. Thus, in these voxels the metrics of bi‐tensor DTI are liable to misestimation, since some signals arising from the fiber bundles not fitted to the single fiber tensor will be considered FW.^34^ Finally, the bi‐tensor model does not account for the non‐Gaussian part of the diffusion decay.^8^

### *Neurite Orientation Dispersion and Density Imaging*

NODDI is a multishell diffusion technique that enables more specific characterization of the tissue microstructure in the whole brain using a clinically feasible protocol.^9^ The NODDI model^9^ assumes that water molecules in neuronal tissue can be considered within three separate compartments: 1) intraneurite space, modeled as restricted diffusion (collection of sticks forming a Watson distribution); 2) extraneurite space, modeled as hindered diffusion (anisotropic Gaussian distribution); and 3) a CSF compartment, modeled as isotropic Gaussian diffusion. The full normalized signal *A* can be written as follows:

(3) $$A=\left(1-V_{\mathrm{iso}}\right)\left(V_{\text{in}}A_{\text{in}}+\left(1-V_{\text{in}}\right)A_{\mathrm{en}}\right)+V_{\mathrm{iso}}A_{\mathrm{iso}}$$

where *A*_*in*_ and *V*_*in*_ are the normalized signal and volume fraction of the intraneurite compartment; *A*_*en*_ is the normalized signal of the extraneurite compartment; and *A*_*iso*_ and *V*_*iso*_ are the normalized signal and volume fraction of the CSF compartment, respectively.

NODDI enables more specific characterization of the tissue microstructure by estimating the packing density of neurite density and the spatial organization or the geometric complexity of the neurites; these are referred to as the neurite density index (NDI) (or intracellular volume fraction [ICVF^15^ or Vic^35^] in other studies) and orientation dispersion index (ODI), respectively—the two key aspects of FA. In contrast with another WM model that shares a common framework, such as WM tract integrity (WMTI), NODDI provides a free water fraction of the isotropic component, referred to as ISO.^36^ The measures of NODDI are able to better delineate WM from GM (the normal WM displays higher NDI and lower ODI, while the reverse is true for GM)^37^ and to differentiate between different GM structures.^38^ Furthermore, ODI has been showed to be strongly correlated with the microglial density; thus, ODI together with ISO have the potential to be the biomarkers of neuroinflammation.^39^

The main limitation of NODDI is the absence of any direct diffusivity estimation. NODDI is predetermined and assumes equal parallel intra‐ and extracellular diffusivity (*D*a,|| = *D*e,|| = 1.7 μm^2^/ms [in adults]).^36^ In addition, the diffusivity of the CSF is fixed at 3 x 10^‐3^ mm^2^/s. Hence, the assumptions underlying NODDI may represent an oversimplification, which could lead to reduced information about the microstructure, and any deviation from these fixed values can bias the remaining parameters, thereby reducing their specificity. Crossing fibers are not explicitly modeled within the NODDI model; consequently, ODI is sensitive to the presence of crossing fibers. In the case where two bundles of fibers cross with only one degenerating, it will exhibit reduced ODI.^40^ Finally, the reproducibility of NODDI measures was shown to be more variable than that of DTI measures; in addition, field strength has a significant effect on NODDI, which calls for careful interpretation of data acquired at 1.5 and 3 T.^14^

## Applications of Diffusion MRI in Neurodegenerative Diseases

### *Alzheimer's Disease*

AlzD is the most common progressive neurodegenerative disorder characterized by gradual memory deficit. The underlying pathological change involves accumulation of amyloid‐β (Aβ) and hyperphosphorylation of tau protein, which leads to the formation of Aβ‐plaques and intracellular neurofibrillary tangles, respectively, leading to neuronal death.^41^ Subjects with no clinical symptoms of AD but who have a parental history of AD or possess a risk gene for AD, the ε4 allele of apolipoprotein E (APOE ε4) or positive CSF tau/Aβ42 biomarkers, are also considered preclinical AD.^42^ Patients with mild cognitive impairment (MCI) are at a higher risk of developing AlzD; moreover, MCI is frequently considered an early stage of AlzD.^43^

MRI volumetry to detect the magnitude and pattern of brain atrophy, notably in the medial temporal lobe including hippocampus, amygdala, and entorhinal area, is now the gold standard for the diagnosis of AlzD.^44^ However, the diagnostic accuracy of brain atrophy for AlzD is only moderately high.^45^ In contrast, diffusion MRI is a potential promising technique for the evaluation of patients with MCI and AD.^46^

#### *DTI in AlzD*

In a multicenter study, significant reduction of FA and a significant increase of MD were demonstrated in core areas of AlzD pathology including corpus callosum, medial and lateral temporal lobes, as well as fornix, cingulate gyrus, precuneus, and prefrontal lobe WM.^47^ Furthermore, in a meta‐analytic study of MCI and AlzD, FA was decreased in all regions except parietal WM and internal capsule, whereas MCI patients had lower FA values in all WM regions except for the parietal and occipital lobes. Increased MD was demonstrated in all WM regions of AlzD patients and in MCI patients in all but the occipital and frontal regions.^48^ Based on the literature,^49^ DTI is a sensitive method for detecting WM changes in patients with MCI and AlzD, who already have widespread impairment of brain region diffusivity. In addition, increasing disease severity is associated with more severe WM disruptions. However, DTI has not been shown to be superior to structural medial temporal lobe volumetry for the detection of early‐stage AD.^49^

#### *DKI in AlzD*

Falangola et al,^50^ for the first time, used DKI to compare groups of healthy controls and patients with MCI and AlzD using manually drawn and automatically generated region‐of‐interest (ROI) analysis. All kurtosis metrics were decreased in the anterior corona radiata of MCI and AlzD patients compared with healthy controls. MK and RK were decreased in temporal oval, segmental temporal, and genu of the corpus callosum, while RK was decreased in segmented prefrontal WM of AlzD patients compared with controls. Compared with healthy controls, MCI showed decreased MK and RK in the prefrontal oval. In studies focused on hippocampus,^51^, ^52^ a significant decrease of MK was demonstrated in MCI and AlzD patients compared with healthy controls, with the lowest value exhibited in AlzD patients. In contrast, FA was similar among the three groups^51^; in addition, no significant difference in hippocampal volume was observed between amnestic MCI and healthy control groups.^52^

**FIGURE 1:**
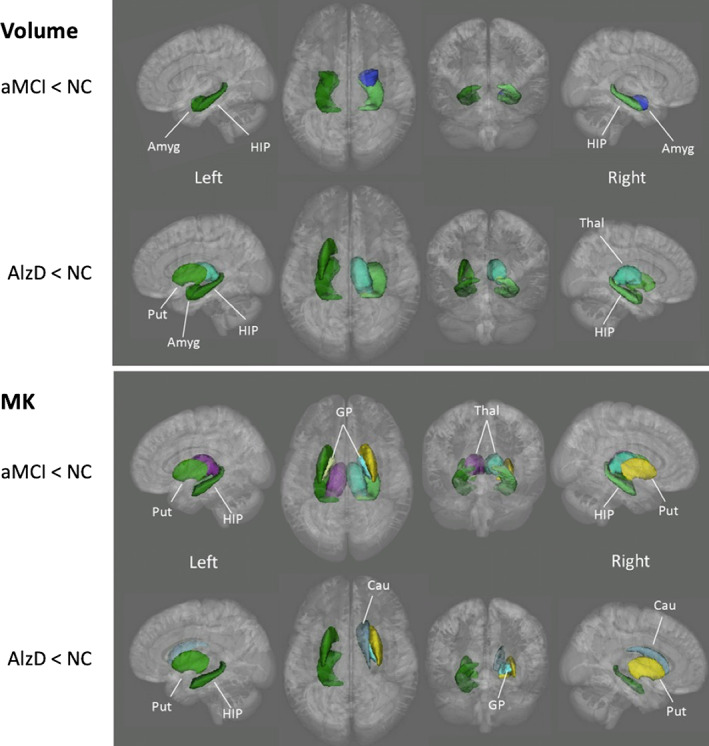
Deep gray matter regions that showed significant differences in volume and mean kurtosis (MK) between healthy controls and patients with amnestic mild cognitive impairment (aMCI)/Alzheimer's disease (AlzD). GP, globus pallidus; Put, putamen; Thal, thalamus; HIP, hippocampus; Cau, Caudate nucleus; Amyg, amygdala. (Adapted and reproduced with permission from Gong et al.^53^)

Gong et al^53^ performed vertex‐wise analysis for cortical GM and ROI analysis for deep GM and observed lower MK in MCI and AlzD patients when compared with healthy controls in all deep GM regions except the amygdala; in addition, the microstructural abnormalities were more broadly distributed compared with the changes in volume and FA (Fig. 1). The changes were believed attributable to the loss of microstructural compartments in AlzD, such as neuronal cell bodies, axons, synapses, and dendrites in cortex and subcortical regions. In contrast, however, MD was the most sensitive metric for capturing cortical microstructural abnormalities in MCI and AlzD patients, especially in the posterior cingulate cortex. The authors speculated that the discrepancy resulted from the microstructural differences between deep GM and cortical GM. Deep GM consists of more densely packed cells with transverse axonal fibers, while cortical GM consists of mainly cell bodies such as astrocytes.^54^

Chen et al^55^ used machine learning to detect WM changes in AlzD; they demonstrated that DKI detected additional abnormalities in the hippocampus and posterior cingulum bundle that were not captured by the DTI indices. However, the combination of DTI and DKI showed a better performance in detecting abnormalities as compared with that of kurtosis or diffusivity alone. Further, in an animal study of AlzD, Vanhoutte et al^56^ demonstrated increased MK, AK, and RK in some ROIs such as the cortex and thalamus of 16‐month‐old APP/PS1 transgenic mouse (a model of cerebral amyloidosis), but no alterations in DTI parameters were observed. The presence of extracellular Aβ plaques has indeed been shown to increase the microstructural complexity of the brain.^56^ Collectively, these studies demonstrated the usefulness of DKI to delineate early microstructural changes in MCI as well as in the late stage of AlzD compared with controls and show the trajectory from controls to MCI to AlzD.

#### *Bi‐tensor DTI in AlzD*

**FIGURE 2:**
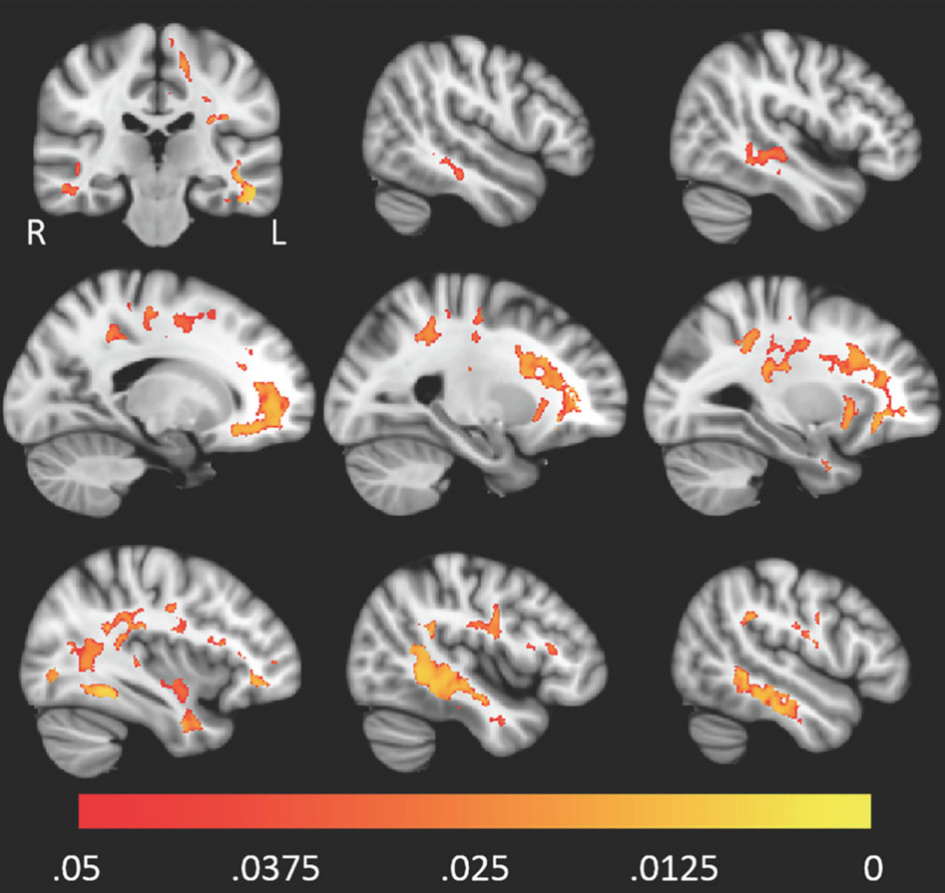
Higher levels of pTau_181_/Aβ42 were associated with higher FW value throughout white matter. The red‐yellow color scale above shows the family‐wise error‐corrected *P*‐value. The underlay image is a T_1_‐weighted MNI template with 1 mm isotropic resolution. (Adapted and reproduced with permission from Hoy et al.^42^)

In a study of patients with preclinical AlzD using voxel‐based analysis, FW derived from bi‐tensor DTI was shown to correlate with the CSF biomarkers of AlzD, such as pTau_181_, Aβ42, YKL‐40, sAPPβ, and tTau, in the bilateral temporal and frontal lobes (Fig. 2).^42^ Among the biomarkers, YKL‐40 is a known marker of microglial activation and neuroinflammation.^42^ Elevated FW has also been found in the hippocampus of patients with MCI compared with controls, while no difference was found with respect to the volume. In addition, FW in the hippocampus is also associated with low CSF Aβ(1‐42) levels and high global amyloid PET values.^57^ A widespread increase in FW in WM of patients with MCI and AD was associated with poorer attention, executive functioning, visual construction, and motor performance. Lower FA_T_ was also shown to be associated with lower memory score in the body of the fornix.^58^

In a study by Dumont et al,^34^ patients with MCI and AlzD exhibited elevated FW in corticospinal tracts and bundles of the limbic system (such as the cingulum and the fornix) compared with healthy controls; in addition, patients with AlzD showed broader pathology as compared with patients with MCI. Higher FW was maintained even after removing GM and CSF partial volume contamination using a WM mask. Significant results were maintained even after removal of WM hyperintensities from the mask, which showed that the between‐group differences with respect to FW metrics were not due to WM lesions.^34^

#### *NODDI in AlzD*

**FIGURE 3:**
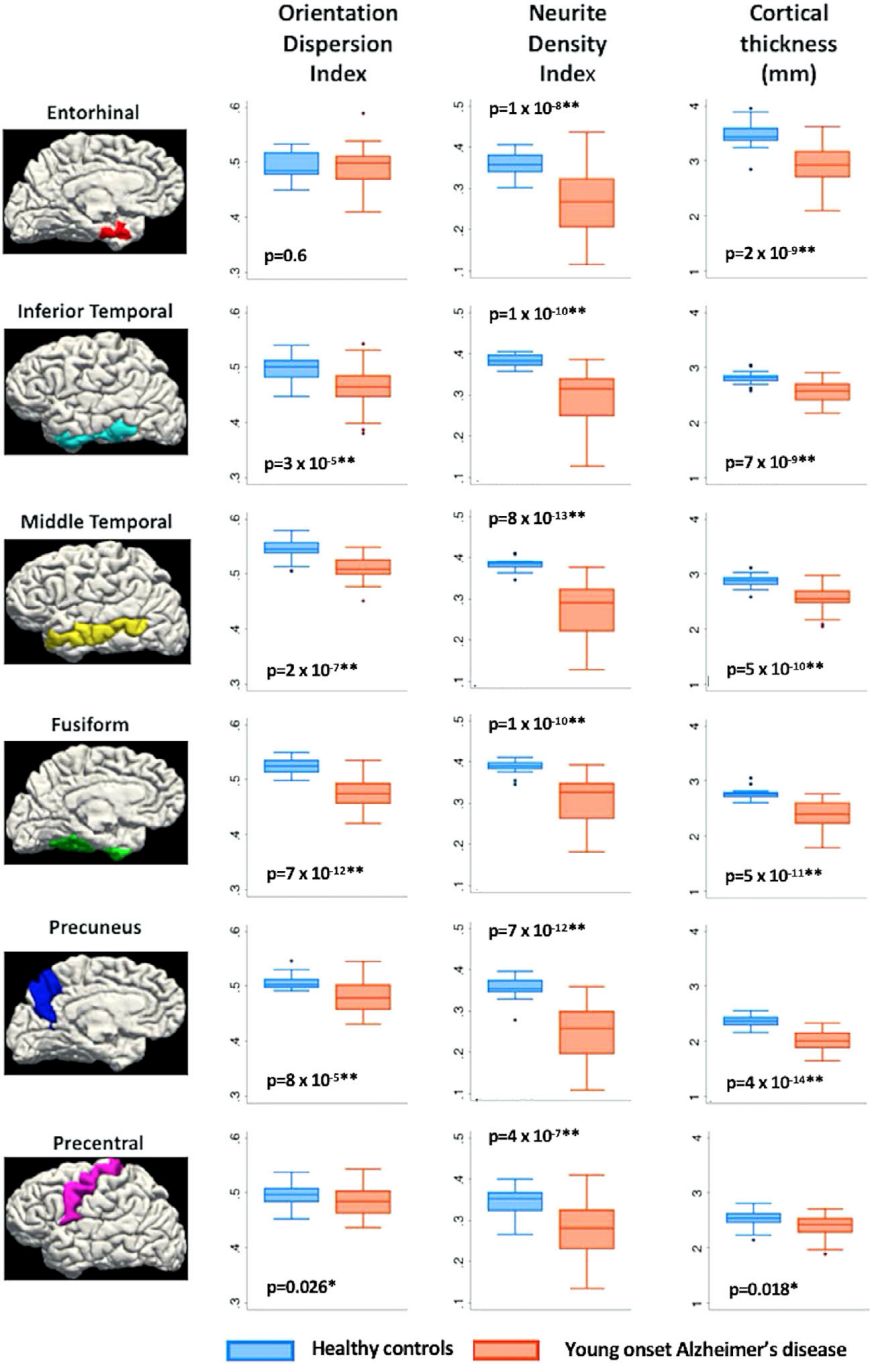
Boxplots of cortical thickness, neurite density index (NDI), and orientation dispersion index (ODI) in healthy controls and patients with early onset of Alzheimer's disease in a priori cortical ROIs: *P < 0.05 **P < 0.008. Bonferroni‐corrected threshold: *P* = 0.05 divided by 6 (total number of ROI). (Adapted and reproduced with permission from Parker et al.^59^)

NDI and ODI were significantly lower in AlzD patients with early onset compared with healthy controls in some predefined ROIs in the cortical GM areas that demonstrated early atrophy in AlzD (entorhinal [only in NDI], inferior temporal, middle temporal, fusiform, and precuneus cortices) (Fig. 3).^59^ Lowered NDI was also demonstrated in the precentral gyrus, an area that is usually relatively spared from atrophy in AlzD with early onset; however, neuropathological studies have shown that the primary motor cortex is vulnerable to significant levels of AlzD‐related pathology.^60^ NDI was shown to exhibit a positive correlation with the results of a Mini‐Mental State Examination; the strongest association was observed in the precuneus, inferior temporal, and middle temporal regions.^59^ Using NODDI and tract‐based spatial statistics (TBSS), WM axonal loss was found more extensive anteriorly in APOE ε4‐positive compared with ε4‐negative AlzD patients with early onset.^61^ Both APOE ε4‐positive and ‐negative AlzD patients with early onset demonstrated lower NDI in WM tracts projecting from the parieto‐occipital lobes (inferior and superior longitudinal fasciculus, inferior fronto‐occipital fasciculus, genu of corpus callosum, and posterior thalamic radiation) compared with healthy controls; however, more widespread changes were observed in ε4‐ positive patients, with additional involvement of the body of the corpus callosum and some parts of the frontal and temporal lobes.^61^ In addition, NDI in WM projections from bilateral parieto‐occipital lobes in all patients showed a correlation with visuospatial and visuoperceptive cognitive performance.^61^

### *Parkinson's Disease*

PD is the second most common progressive neurodegenerative disorder that involves multiple neurotransmitter pathways that are associated with a range of clinical features. The diagnosis of PD is based on the presence of motor deficit including bradykinesia, rigidity, and tremor. The motor features result from a selective loss of dopaminergic neurons in the substantia nigra (SN) pars compacta, and widespread aggregation of α‐synuclein immunoreactive inclusions in the form of Lewy pathology comprising Lewy neurites and Lewy bodies.^62^, ^63^ Histopathological studies have shown that up to 70% of dopamine neurons may be lost by the time of the initial diagnosis of PD.^64^ Thus, identification of sensitive biomarkers of PD is a key imperative.

#### *DTI in PD*

The parameters of DTI, especially FA and MD, were able to distinguish between PD patients and healthy controls. According to recent meta‐analytic studies,^65^, ^66^ patients with PD consistently exhibit decreased FA and/or increased MD in the SN, corpus callosum, frontal lobe, and the cingulate and temporal cortices. However, Guimarães et al^67^ questioned the ability of DTI to detect WM changes in early PD. They assessed early, moderate, and severe PD using DTI and found significant abnormalities only in the severe PD group.

#### *DKI in PD*

**FIGURE 4:**
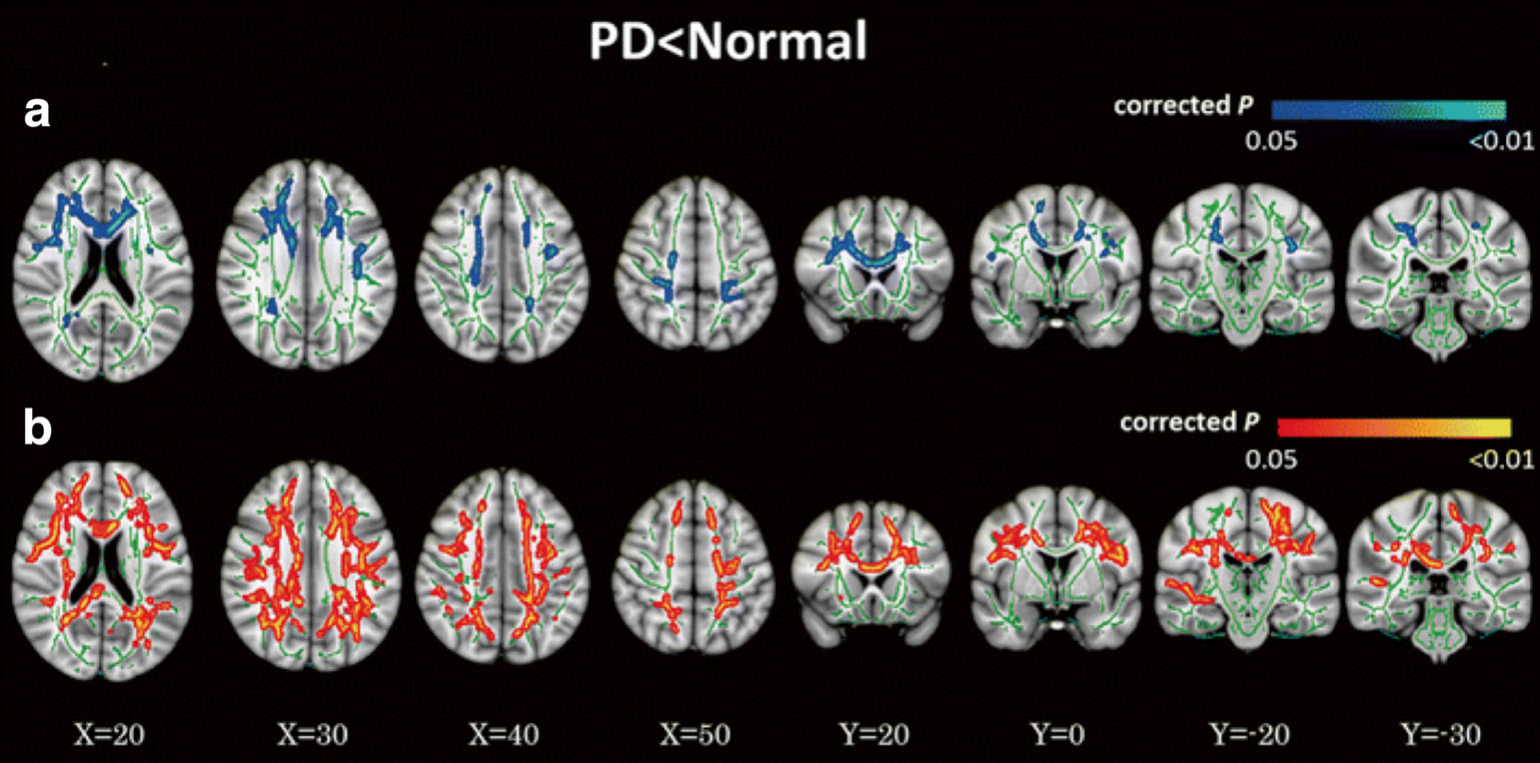
Comparison of DTI and DKI metrics between patients with Parkinson's disease (PD) and healthy controls. Tract‐based spatial statistics (TBSS) maps of decreased fractional anisotropy (FA) **(a)** and decreased mean kurtosis (MK) **(b)** in PD patients compared with age‐matched healthy subjects shown in neurological convention. In the TBSS maps, the FA skeleton with FA >0.2 is shown in green; voxels in which the one‐sided permutation‐corrected *P* was <0.05 are marked in blue (FA) or red (MK). (Adapted and reproduced with permission from Kamagata et al.^71^)

Only two studies have evaluated the SN in PD using DKI and both studies found a significant increase in MK.^68^, ^69^ Kamagata et al^70^, ^71^ performed several studies to evaluate the WM of PD patients using DKI. In the first study using tract‐specific analysis, MK and FA were found to be decreased in the anterior cingulum in PD patients, while MK showed better diagnostic performance. The anterior cingulum is the part of the brain that shows early pathological changes in PD; therefore, these findings support the use of DKI as an early diagnostic biomarker of PD.^70^ Furthermore, using TBSS analysis, Kamagata et al^71^ demonstrated that DKI is a more sensitive modality than DTI for detecting WM changes in PD patients; in contrast, a reduction in MK values occurred more extensively throughout the brain (such as in the frontal, parietal, and occipital WM, and corpus callosum) as compared with FA reduction in patients with PD (Fig. 4). Reduced MK was also demonstrated in areas with crossing fibers (such as corona radiata and SLF), whereas FA did not show any changes in these areas.^71^ Kamagata et al^15^ also evaluated the GM using DKI and NODDI, which is further discussed in a later subsection (NODDI in PD).

#### *Bi‐tensor DTI in PD*

FW within the SN is considered a promising biomarker to distinguish patients with PD from healthy controls and as a biomarker of disease progression in PD. Both single‐ and multisite studies comprising large cohorts have shown that PD patients have higher FW and unchanged FA_T_ values in the posterior SN compared with healthy controls.^72^, ^73^, ^74^ Moreover, FW values in the posterior SN showed a correlation with the severity of motor symptoms.^72^ In a study, patients with long‐standing PD exhibited increase in FW in the anterior and posterior SN, which was considered attributable to posterior‐to‐anterior SN degeneration.^75^ FW may also be used to differentiate PD from atypical parkinsonism diseases, such as multiple system atrophy (MSA) and progressive supranuclear palsy (PSP). Planetta et al^74^ showed increased FW in the SN in all forms of parkinsonism. However, both MSA and PSP, but not PD, exhibited widespread elevated FW and altered FA_T_ beyond the SN, including in the basal ganglia, thalamus, and cerebellum.^74^

In longitudinal studies, FW in the posterior SN was shown to increase with the progression of PD over 1 year^72^ and 4 years,^76^ whereas free water values did not change in healthy controls. One‐year and 2‐year longitudinal changes of FW can be used to evaluate the progression using the Hoehn and Yahr staging system.^76^ In addition, increased FW in the posterior SN has been shown to be associated with a higher Hoehn and Yahr scale, MDS‐UPDRS‐III total motor scores, postural and gait and tremor scores.^77^ Moreover, increased FW in caudate and posterior SN were associated with higher dementia ratings.^77^ Inverse correlations were also found between FW in posterior SN and vesicular monoamine transporter type 2 (VMAT2) binding (reflecting diminished nigrostriatal dopaminergic nerve integrity) in putamen and caudate.^77^

**FIGURE 5:**
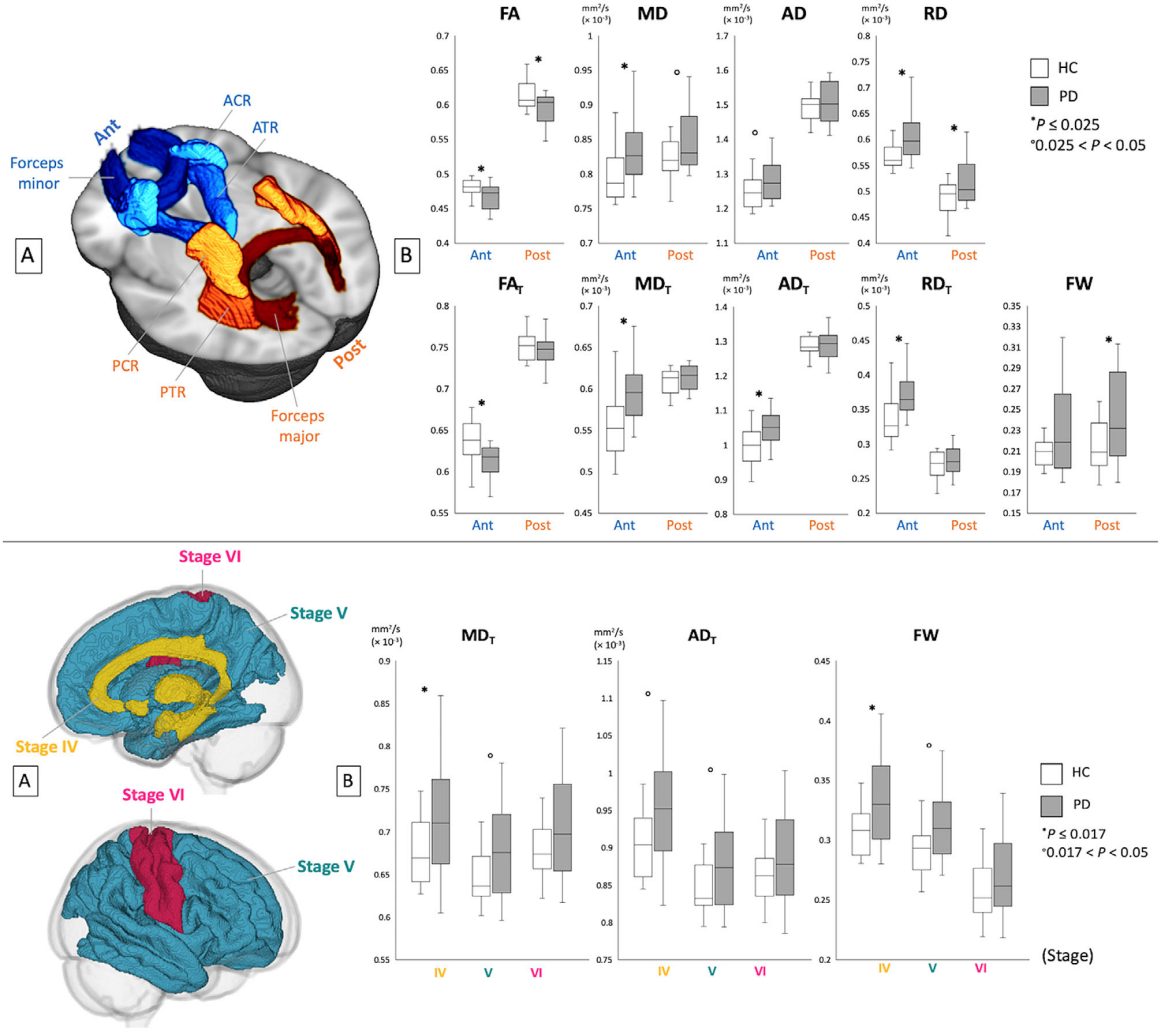
Upper panel **(a)** ROI analyses of the anterior (ACR, ATR, and forceps minor) and posterior (PCR, PTR, and forceps major) white matter areas. **(b)** Mean values for DTI (FA, MD, AD, and RD) and FW imaging (FA_T_, MD_T_, AD_T_, RD_T_, and FW) indices of the anterior and posterior white matter areas in healthy controls (HC; white bars) and patients with Parkinson's disease (PD) (gray bars). Lower panel (a) ROI analyses of gray matter areas belonging to Braak stages IV, V, and VI. (b) Mean values for FW imaging indices (MD_T_, AD_T_, and FW) of each area in healthy controls (HC; white bars) and patients with PD (gray bars). ACR, anterior corona radiata; ATR, anterior thalamic radiation; PCR, posterior corona radiata; PTR, posterior thalamic radiation. (Adapted and reproduced with permission from Andica et al.^30^)

The FW elimination technique has also been used to evaluate the WM and GM of PD patients using TBSS, GM‐based spatial statistics (GBSS), and ROI analyses. The changes in bi‐tensor DTI indices were demonstrated in somewhat more specific WM areas compared with the changes of DTI indices, whereas lower FA_T_ and higher MD_T_, AD_T_, and RD_T_ (indices of neuronal degeneration) in anterior WM areas as well as higher FW (index of neuroinflammation) in posterior WM areas were observed compared with the controls (Fig. 5). The author assumed that these finding are in line with the fact that neuroinflammation precedes axonal degeneration in PD.^30^ Patients with PD showed higher MD_T_, AD_T_, and FW in GM areas corresponding to Braak stage IV, while there was no significant difference in conventional DTI measures (Fig. 5). This suggests that the FW imaging indices are more sensitive for detection of GM abnormalities in patients with PD.^30^

#### *NODDI in PD*

PD patients were shown to exhibit decreased NDI in the contralateral SN pars compacta and putamen compared with healthy controls. On receiver operating characteristics curve analysis, NDI also showed the best diagnostic performance compared with DTI measures.^78^ Another study revealed decreased NDI in the contralateral distal part of the nigrostriatal pathway in PD patients, which may reflect retrograde degeneration; however, no changes were observed in DTI parameters.^35^

**FIGURE 6:**
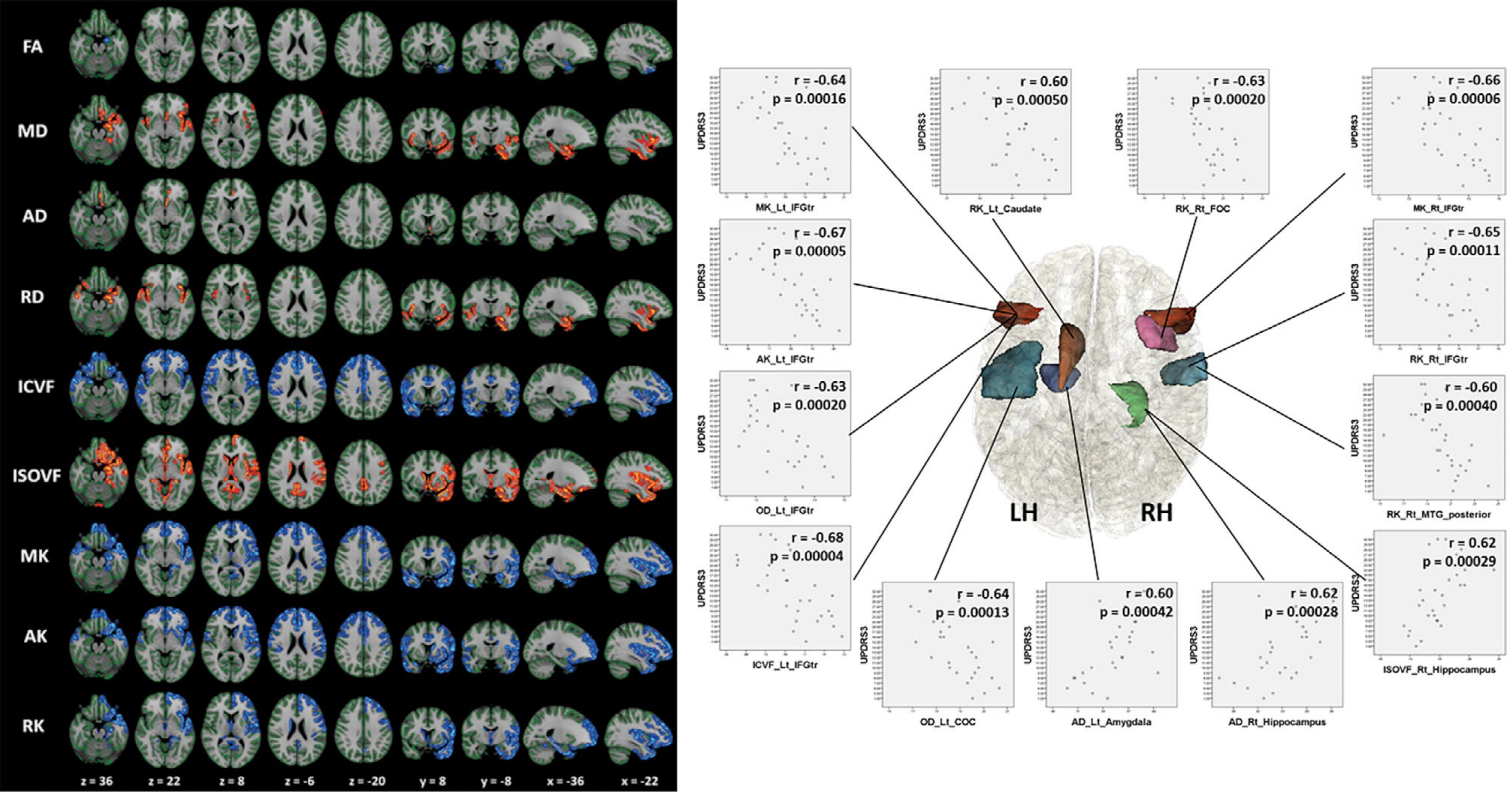
Left panel: Comparison of DTI, DKI, and NODDI metrics between Parkinson's disease (PD) patients and controls. Gray matter based spatial statistics (GBSS) results showed reduced FA, ICVF, MK, AK, and RK (blue‐light blue voxels), and increased MD, AD, RD, and ISOVF (red‐yellow voxels) in PD patients when compared with age‐matched healthy subjects. All images are displayed in Montreal Neurological Institute space using neurological convention. In patients with PD, cortical GM in the frontal, temporal, limbic, and paralimbic areas exhibited significantly reduced MK, AK, RK, and ICVF when compared with the control group (GBSS analysis). Regions where significant changes in the conventional DTI parameters (FA, AD, and RD) occurred were noticeably smaller than those where significant changes in MK, AK, RK, and ICVF were observed. To aid visualization, the results (corrected *P* < 0.05) are thickened using the fill script implemented in FSL. Right panel: Regions where dMRI parameters were significantly correlated with the Unified Parkinson's Disease Rating Scale (UPDRS)‐III‐motor subscale scores in the PD group using ROI analysis; scatter diagrams of these regions. MK, AK, ICVF, and OD of the left inferior frontal gyrus pars triangularis (IFGTr); MK and RK of the right IFGTr, OD of the Lt central opercular cortex (COC); RK of the right frontal opercular cortex (FOC); RK of the right middle temporal gyrus posterior division; and RK of the left caudate showed a significant negative correlation with UPDRS‐III scores. ISOVF and AD of the right hippocampus and AD of the left amygdala (AMY) showed a positive correlation with UPDRS‐III scores. (Adapted and reproduced with permission from Kamagata et al.^15^)

In a study^15^ evaluating DKI and NODDI in the GM using GBSS and ROI analyses, DKI (decreased MK, AK, and RK) and NODDI (reduced NDI and increased ISO) parameter changes were observed in the cortices of frontal, temporal, limbic, and paralimbic areas that corresponded to Braak stages IV and V, when compared with the healthy controls (Fig. 6). The authors suggested that these changes may reflect the sparse neurite structure and neuronal loss caused by inhibition of neurite outgrowth and branching inhibition in the GM. Although PD patients displayed DTI parameters changes, such as decreased FA and increased MD, AD, and RD, the abnormalities were more circumscribed when compared with the more widespread abnormalities identified with the DKI and NODDI parameters (Fig. 6). Thus, DKI and NODDI appear to be more sensitive than the DTI parameters for the detection of GM abnormalities in PD. This was reinforced by the findings of linear discriminant analysis, which showed that MK and NDI maximized the predictive accuracy of the diagnosis. In addition, the changes in DKI and NODDI parameters in the frontal, temporal, basal ganglia, limbic, and paralimbic areas were shown to correlate with UPDRS‐III scores, reflecting the severity of motor impairment.

### *Amyotrophic Lateral Sclerosis*

ALS is a neurodegenerative disease primarily characterized by progressive atrophy and weakness of the limbs, as well as the bulbar and respiratory muscles due to the impairment of lower and upper motor neurons.^79^ The pathogenesis of ALS remains largely unknown; however, repeat expansions in the chromosome 9 open reading frame 72 gene (*C9orf72*) are the most commonly known genetic causes of ALS. The diagnosis of ALS is based on a history of painless progressive weakness associated with signs of upper and lower motor dysfunction.^79^ To date, there is no definitive diagnostic test for ALS; therefore, there is a need to identify noninvasive neuroimaging biomarkers.

#### *DTI in ALS*

According to some systemic reviews and meta‐analytic studies,^5^, ^80^, ^81^, ^82^ FA is consistently reduced in the corticospinal tract and posterior limb of the internal capsule of patients with ALS; in addition, it is often accompanied by increased MD, RD, or AD. Decreased FA was also found in the posterocentral portion of corpus callosum, which is known to contain fibers connecting the two motor cortices.^83^, ^84^ Additional areas within the frontal, temporal, and parietal areas have shown reduced FA. These findings confirm the notion that ALS is a multisystem disease involving both motor and extramotor regions. Some longitudinal studies have found a reduction in FA in the CST, whereas others have not.^80^ Overall, DTI seems to be a promising diagnostic biomarker of ALS; however, the sensitivity and specificity are relatively low (0.65 and 0.67, respectively).^85^

#### *DKI in ALS*

In the ROI analysis of the motor cortex (contralateral to the symptomatic limbic) of ALS patients with mild‐to‐moderate impairment, MK, AK, and RK were found to be reduced compared with that in healthy controls; however, no significant between‐group differences were observed with respect to the DTI parameters.^86^ RK was also associated with the ALS functional rating scale revised version (ALSFRS‐R), which is the disease severity score for ALS.^86^

**FIGURE 7:**
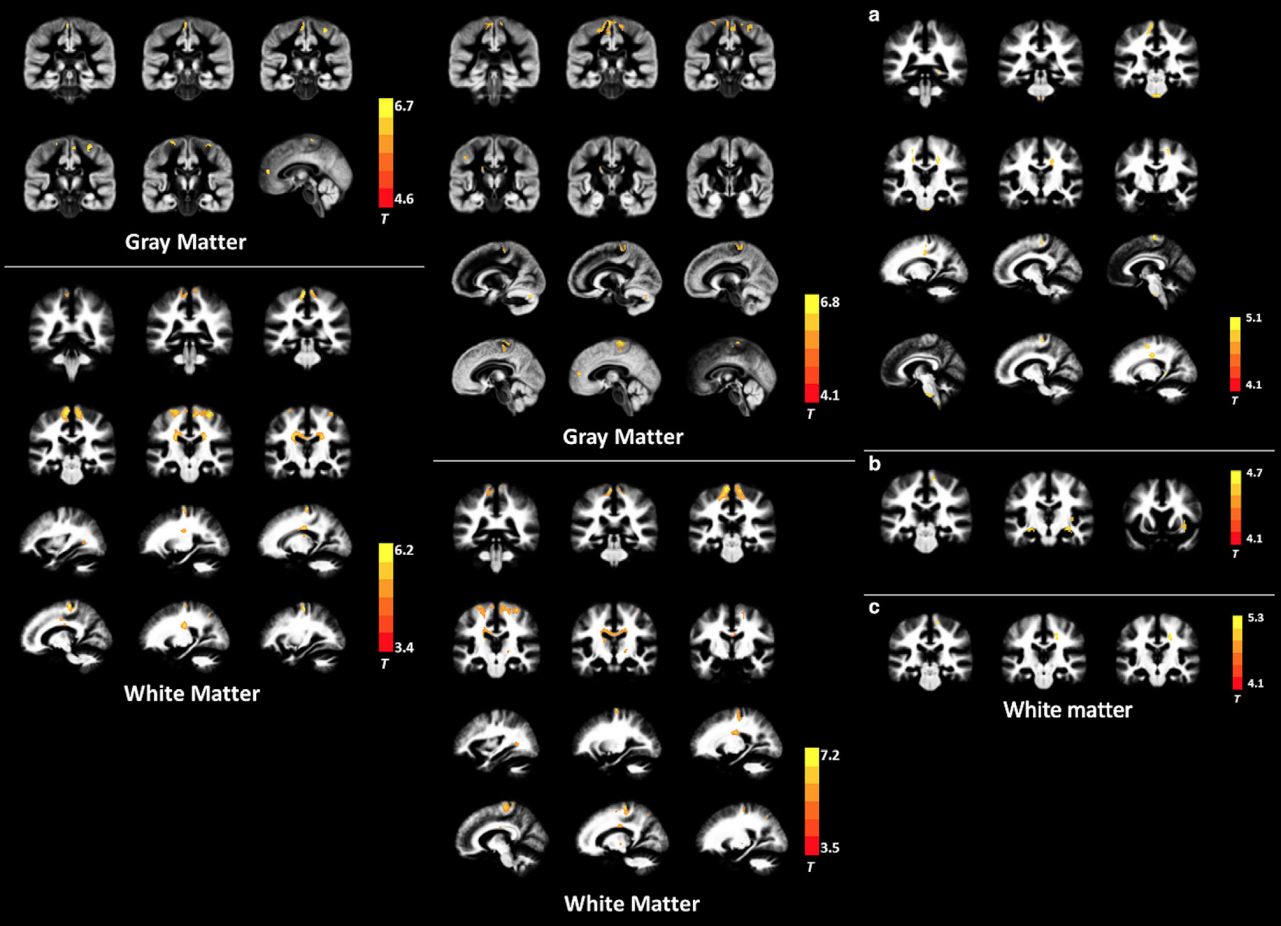
Left and middle panel: Gray and white matter regions with significantly decreased MK in amyotrophic lateral sclerosis (ALS) patients compared with healthy controls. Middle panel: Gray matter and white matter regions with significantly decreased RK in ALS patients compared with healthy controls. Right panel: White matter regions with significantly decreased FA **(a)**, increased MD **(b)**, and increased RD **(c)** in ALS patients compared with healthy controls. The images displayed are overlaid on the averaged WM and GM maps from all subjects. (Adapted and reproduced with permission from Huang et al.^87^)

In a voxel‐based analytic study, ALS patients showed lower MK and RK in the following WM areas as compared with that in controls: bilateral precentral gyrus, bilateral corona radiata, bilateral middle corpus callosum, left occipital lobe, and right superior parietal lobule. In the GM, ALS patients showed decreased MK and RK in the bilateral precentral gyrus, bilateral paracentral lobule, and left anterior cingulate gyrus (Fig. 7).^87^ Reduced FA and increased MD and RD were also found in the WM of patients with ALS; however, the spatial extent was smaller (Fig. 7).^87^ In the same study, among the WM regions, the MK values of right WM precentral gyrus showed a positive correlation with the ALSFRS‐R score, while MK and RK values in the left precentral gyrus showed a negative correlation with disease duration. Among the GM regions, the RK values in the left caudate body showed a positive correlation with the ALSFRS‐R score.^87^ Interestingly, in both studies,^86^, ^87^ reduced RK was consistently demonstrated in patients with ALS, while reduced AK was only found in one study. RK is believed to reflect myelin integrity; thus, RK reduction may indicate the impairment of myelin in ALS. Indeed, postmortem studies have demonstrated demyelination in patients with ALS.^88^

#### *Bi‐tensor DTI in ALS*

To the best to our knowledge, no studies have investigated the use of bi‐tensor DTI in ALS.

#### *NODDI in ALS*

**FIGURE 8:**
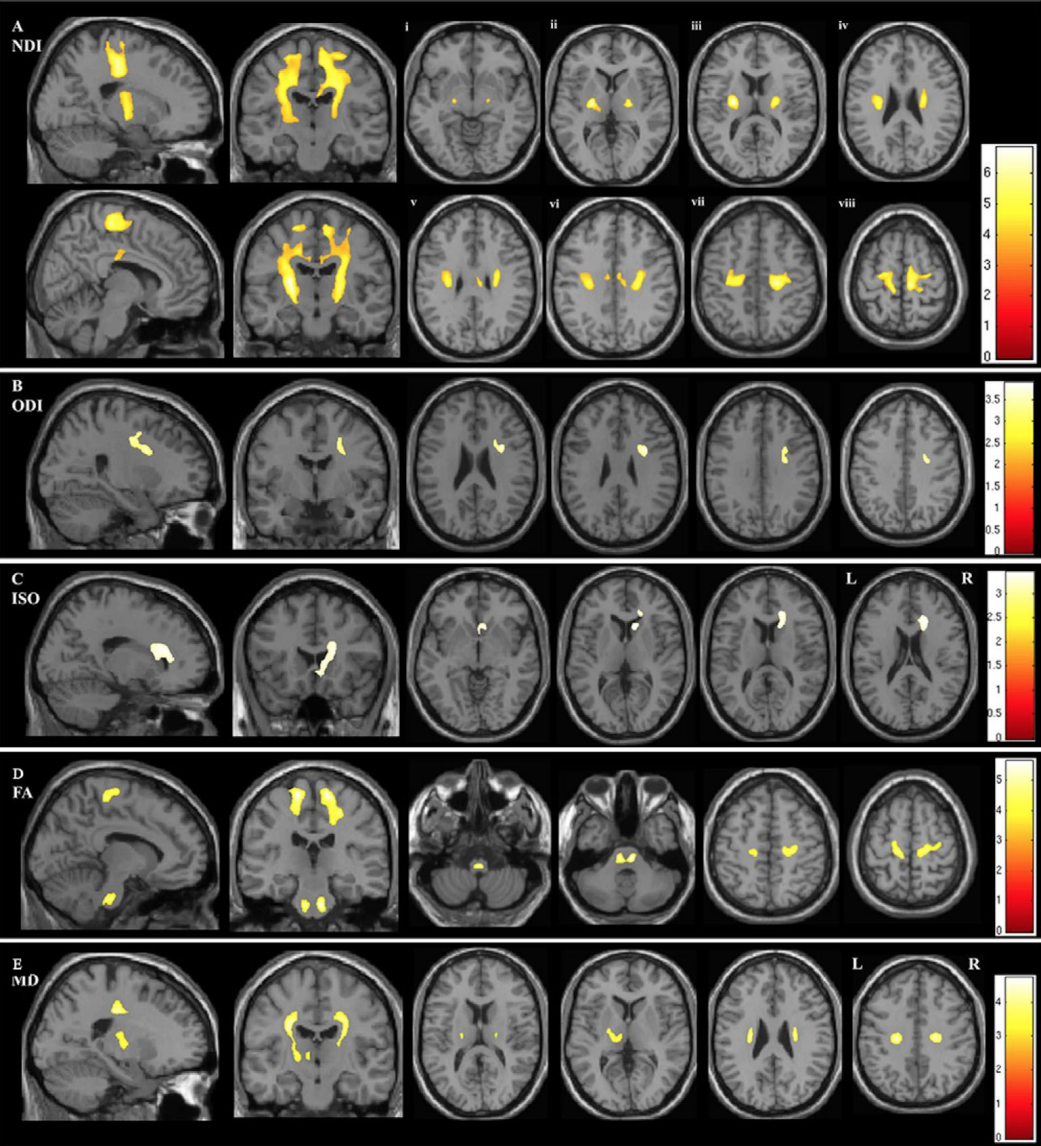
The areas of significant difference between the amyotrophic lateral sclerosis (ALS) and control groups on whole brain analysis of the NODDI parameters **(a)** NDI, **(b)** ODI and **(c)** ISO and DTI parameters **(c)** FA and **(e)** MD. The results are shown using a statistical significance of *P* < 0.05 after family‐wise error correction at the cluster level; clusters formed using *P* < 0.001. Figures Ai–Aviii demonstrate the areas of significant difference in NDI on axial sections from the posterior limb of the internal capsule (vi) extending rostrally up into the subcortical WM of the precentral gyrus (viii). (Adapted and reproduced with permission from Broad et al.^89^)

Whole‐brain voxelwise analysis in patients with ALS manifestation using NODDI demonstrated significantly reduced NDI throughout the intracranial corticospinal tracts up to the subcortical WM of the precentral gyri and across the corpus callosum, with increased ODI in the anterior limb of right internal capsule and increased ISO adjacent to the right lateral ventricle relative to healthy controls (Fig. 8).^89^ Further, decreased NDI was observed within the right corona radiata and precentral subcortical WM to a greater extent in patients with both limb and bulbar involvement compared with those with limb involvement alone.^89^ In this study, longer disease duration showed a correlation with reduced ODI in the precentral gyri, dorsolateral prefrontal cortices, and precuneus.^89^ As expected, FA was reduced within the corticospinal tracts but less extensive compared with NDI; these findings showed that NODDI may be more sensitive than DTI (Fig. 8).^89^

ROI analysis showed that NODDI also had greater sensitivity compared with DTI in the WM and GM volumetry of presymptomatic carriers of the *C9orf72* mutation.^90^ Compared with noncarriers, *C9orf72* mutation carriers demonstrated WM alterations in 10 tracts, involving frontotemporal‐related and corticospinal tracts with NDI and only five tracts with DTI (increased MD, AD, and RD) metrics. Effect size results confirmed that NDI was more sensitive than DTI metrics, whereas the effect size of the two tracts was significantly higher for NDI than for DTI metrics.^90^ Further, altered cortical regions were demonstrated with increased ISO in 13 regions, whereas 11 regions displayed volumetric atrophy.^90^ Collectively, both studies suggested that WM integrity abnormalities in ALS are mainly caused by neuron loss.^89^, ^90^

### *Huntington's Disease*

HD is an autosomal dominant, progressive neurodegenerative disorder that typically presents in young middle age. The condition is characterized by motor, cognitive, and psychiatric disturbances.^91^ The abnormal expansion of cytosine‐adenine‐guanine repeats in the Huntington gene has been shown to cause a selective loss of medium spiny neurons, especially in the striatum; however, this is followed by reduction in WM surrounding the basal ganglia, which extends to cortical WM throughout the cortex.^91^, ^92^ Owing to the knowledge of this gene mutation, HD is one of the rare neurodegenerative conditions for which predictive genetic testing is available for individuals with a known family history. This enables identification of HD gene mutation carriers or presymptomatic HD (pre‐HD). However, structural MRI has also been shown to be useful for evaluation of pre‐HD. Volume loss, notably in the striatum, is detectable 1–2 decades prior to the development of motor features in HD.^93^ The atrophy progresses over time^94^ and correlates with disease load.^95^ This supports the use of structural MRI as a state biomarker in HD; however, it has its limitations, as it does not provide a direct pathological measure of disease.

#### *DTI in HD*

A recent meta‐analysis^96^ including 140 pre‐HD, 235 symptomatic HD (sym‐HD), and 302 controls showed DTI parameters abnormalities in the basal ganglia and corpus callosum of patients with HD. Specifically, both pre‐HD and sym‐HD patients showed significantly increased FA in the caudate, putamen, and globus pallidus and significantly decreased FA in the corpus callosum as compared with that in controls. In addition, significantly increased MD was demonstrated in the putamen and thalamus of both pre‐HD and sym‐HD, and in the caudate of sym‐HD patients, compared with controls. In the corpus callosum there was a significant increase of RD and AD in sym‐HD patients compared with controls. In a longitudinal study that compared patients with HD (including pre‐HD and early sym‐HD) and healthy controls over a 1‐year period, a significant reduction of FA that overlapped with declining AD between baseline and 1‐year follow‐up were demonstrated within subcortical, callosal, and frontrostriatal tracts, including the ascending limb of the internal capsule and the superior corona radiata.^97^

#### *DKI in HD*

**FIGURE 9:**
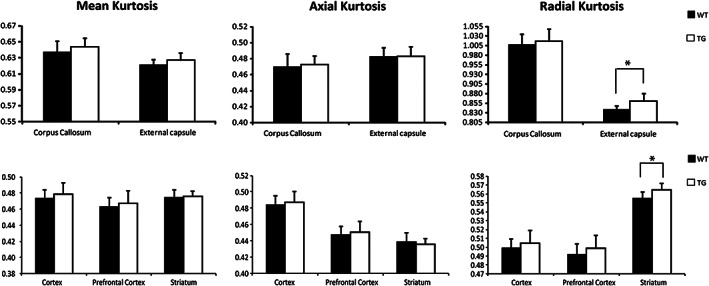
Mean and standard deviation of diffusion parameters measured in different ROIs (gray matter: (pre)frontal cortex, cortex, and striatum–white matter: corpus callosum and external capsule) in tgHD rats and Wt littermates. RK was increased in tgHD rats in the striatum and external capsule. *P < 0.05. (Adapted and reproduced with permission from Blockx et al.^98^)

To date, DKI has not been applied in patients with HD. However, in some studies using ROI analysis, kurtosis indices have been shown to change with the pathological alterations in aged transgenic HD rats. Blockx et al^98^ demonstrated an increase of RK in the striatum and external capsule and suggested that the changes in the striatum appear to be due to a high degree of diffusion complexity and restriction, while the changes in the external capsule reflect fiber composition or cell permeability (Fig. 9). Histologically, RK showed a positive correlation with glial fibrillary acidic protein, which is expressed by the astrocytes in the striatum.^98^ Another study assessed the use of DKI for the assessment of WM and GM in developing transgenic HD rat pups at postnatal days 15 and 30. AK values in the caudate and putamen at postnatal day 30 were higher than that in the controls.^99^ Both studies indicated that DKI is a sensitive method for detecting HD‐associated WM and GM abnormalities.

#### *Bi‐tensor DTI in HD*

Steventon et al^100^ performed bi‐tensor DTI and measured the tissue volume fraction (TVF), which reflects the estimated fractional volume of tissue after FW elimination, in the corpus callosum of patients with HD using ROI and tractography approaches. They demonstrated reduced TVF in patients with HD compared with healthy controls; in addition, TVF was found to be a more sensitive parameter of disease burden compared with DTI metrics. Reduction of TVF along the WM pathways is suggestive of reduced packing density in the tissue, which may be due to a loss of axons or demyelination.^100^

#### *NODDI in HD*

**FIGURE 10:**
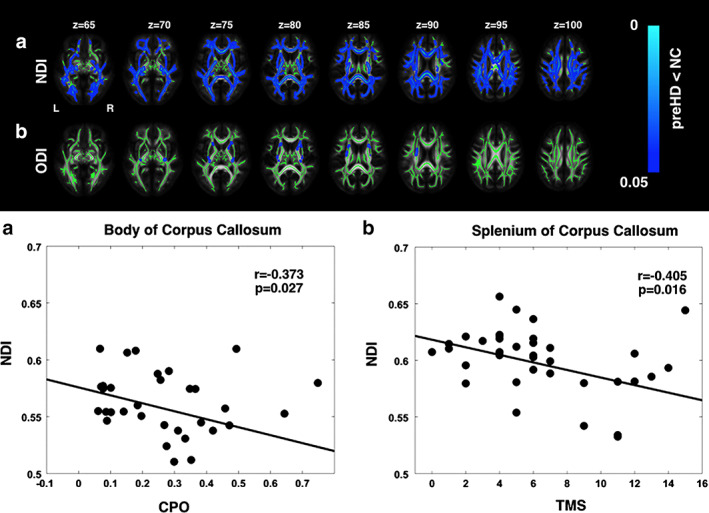
Upper panel: The regional distribution of differences in NODDI parameters in premanifest Huntington's disease (pre‐HD) gene carriers compared with controls (NC). There were reductions in neurite density (NDI) across the whole brain, indicating a reduction in axonal density **(a)**, as well as localized reductions in the dispersion of fibers (ODI) in the corpus callosum and the internal and external capsule, indicating select pruning of white matter fibers **(b)**. Threshold‐free cluster enhancement *P* < 0.05. Group differences in NODDI metrics are overlaid on white matter skeleton. Lower panel: Correlation of NDI with clinical markers of disease progression. (a) Negative correlation between NDI in the body of the corpus callosum and cumulative probability to onset. (b) Negative correlation between NDI in the splenium of the corpus callosum and total motor score. (Adapted and reproduced with permission from Zhang et al.^92^)

In a study of premanifest HD (pre‐HD) using NODDI, a widespread reduction in axonal density (indexed by NDI), which overlapped with increased MD, was observed in the WM tracts, including the corpus callosum and the surrounding basal ganglia; reduced NDI in the corpus callosum showed a positive correlation with a marker of severity (Fig. 10). The results of TBSS and ROI analyses suggested that axonal pathology is a major factor underlying WM degeneration in pre‐HD.^92^ Moreover, increased ODI, an indicator of increased axonal organization, was demonstrated in the tracts surrounding the basal ganglia and in the internal and external capsule compared with controls; this reflected potential compensatory pruning of axons.^92^

## Conclusion and Future Directions

Early diagnosis of neurodegeneration is important for the future development of neuroprotective therapies for neurodegenerative diseases. This review indicated that DKI, bi‐tensor DTI, and NODDI may serve as potential sensitive biomarkers for assessment of microstructural changes in neurodegenerative diseases and as biomarkers of disease progression. Additionally, DKI, bi‐tensor DTI, and NODDI showed remarkable advantages over DTI with respect to detection of GM changes in neurodegenerative diseases.

Importantly, the FW map obtained with bi‐tensor DTI and NODDI may be used as a biomarker of neuroinflammation.^30^, ^31^, ^39^ The role of neuroinflammation in neurodegeneration must also be fully elucidated, since proinflammatory agents have been widely detected in patients with neurodegenerative diseases.^101^ Furthermore, NODDI may provide biomarkers of neurite density and orientation dispersion.^9^ Indeed, neurodegeneration is associated with chronic progressive loss of the structure of neurons.^101^ However, considering the limitations of each technique, the interpretation of changes in the diffusion indices is complex and should be performed with caution.

A growing body of evidence suggests the involvement of myelin as a crucial neuropathological feature of neurodegenerative diseases.^102^ As diffusion MRI is insensitive to myelin, simultaneous evaluation using diffusion MRI and a myelin imaging technique is required to demonstrate a more complete picture of neuropathology of neurodegenerative diseases.

Even though multiple sclerosis has been recently considered a neurodegenerative disease, it was not covered in the current article.^103^ Furthermore, we did not discuss the usefulness of other more complex diffusion MRI microstructural models such as composite hindered and restricted model of diffusion or AxCaliber^104^ in neurodegenerative diseases, mainly because their clinical application in patients with movement disorders is limited by the long duration of the scanning protocols. However, these topics warrant further discussion.
